# Biological pest regulation can benefit from diverse predation modes

**DOI:** 10.1098/rsos.240535

**Published:** 2024-09-18

**Authors:** Deyatima Ghosh, Amaël Borzée

**Affiliations:** ^1^ Laboratory of Animal Behaviour and Conservation, College of Life Sciences, Nanjing Forestry University, Nanjing, Jiangsu 210037, People’s Republic of China; ^2^ Jiangsu Agricultural Biodiversity Cultivation and Utilization Research Center, Nanjing 210014, People’s Republic of China

**Keywords:** amphibians, foraging mode, pest management, reptiles, sustainable agriculture

## Abstract

Increases in agricultural intensity due to anthropogenic demands alongside the need to reduce the reliance on pesticides have resulted in an urgent need for sustainable options for pest control. Biological pest regulation is an alternative strategy that relies on natural predators and is essentially a by-product of successful foraging. Therefore, knowledge of the predator’s specific foraging behaviour can significantly improve bioregulation. In this article, we discuss the implications of predators’ diverse foraging modes on their efficiency as bioregulators of crop pests using amphibians and reptiles as models. Amphibians and reptiles are promising bioregulators as they are insectivorous, and the diversity in their foraging styles—ambush and active foraging, differing in energy expenditure, movement, cognitive abilities, reliance on cues, response to predatory risk, competition and prey salience—can have specific impacts on pest regulation. We propose the uptake of this concept into strategizing pest management actions. We are now moving towards an era of biological pest regulation, which is the most targeted, economically profitable method with zero negative impact on the ecosystem. Utilizing diverse traits associated with the different foraging modes in vertebrate predators can be a crucial tool in allowing pest management to adapt to the extreme challenges it is facing.

## 1. Introduction

Agricultural intensification has not only resulted in the loss of biodiversity and key ecosystem services but has also resulted in global challenges in mitigating crop pests [1]. For six decades, using agrochemicals has been the conventional strategy against pests; however, the detrimental impacts of chemical pesticides on biodiversity, crop quality and human health are now evident, and it is pertinent to look for a sustainable solution, such as biological pest regulation [2]. Biological pest regulation controls crop pests either through direct predation by pest predators or competition for resources inhibiting the growth and spread of pests, reducing the population below the threshold to prevent economic impact [3]. To date, biological pest regulation is dependent on arthropods due to their short life cycle and temporal synchronicity with crop pests. Though primarily an ecological tool, biological pest management strategies have effectively used behavioural manipulation in crop pests and arthropod pest predators. Techniques such as utilizing pheromones to alter oviposition sites, disrupt mating and change foraging preferences of the pests have shown success [4]. However, arthropod pest predators interact with the environment and ecosystem in their niche due to their limited dispersal ability [5]; thus, restricting the spatial scales at which interventions can occur, which are different from vertebrates [5]. Additionally, food sources vary in nutritional quality, quantity and distribution across seasons and over time, requiring animals to rapidly adapt to these uncertainties through their foraging abilities. Vertebrates exhibit a wide range of behavioural flexibility associated with foraging, including prey preference, patch switching, response to prey salience, quantity and quality judgement, spatial learning and retaining information from previous experiences [6]. These traits enhance their value as pest predators, offering significant potential for long-term, sustainable pest management. While arthropods are effective bioregulators, including vertebrates into bioregulation strategies can greatly improve efficiency. To achieve more effective and sustainable pest management, it is essential to leverage the strengths of vertebrate predators alongside arthropod bioregulators [7]. Vertebrates such as birds, bats, amphibians and reptiles reduce pests in tropical crops such as coffee and cacao as well as orchards, vegetables and paddy crops [8,9]. Amphibians and reptiles make efficient pest regulators as most species are generalists, insectivorous (reptiles also feed on vertebrate mammal pests), feed on a wide range of crop pests and can target both ground-dwelling pests and pests in flight or at the surface of crops [10]. Their importance in pest management has been recognized in diverse crops including rice, vegetables and coffee [9–13]. Artificial introduction of frogs in paddy fields has shown a significant increase in yield, an improvement in the biochemical properties of crops and soil and a 60% reduction in crop pests [14,15]. Experimentally, reptiles have also been shown to reduce pests between 49 and 84% [11]. In Brazil alone, pest control by anurans accounts for $1.18 billion for all crops and they have the potential to reduce 300 million crop pests annually [16,17]. However, what makes them unique, hence more promising, is the presence of two foraging modes that can further influence their foraging behaviour, thus their pest regulatory role [18]. Therefore, their behavioural flexibility and rapid adaptability to changing environments make them ideal candidates for enhancing pest control efforts.

## 2. Link between biological pest regulation and the foraging mode of predators

To date, biological pest regulation has been used as a tool for ecosystem management [19]. However, the existing approaches to mitigate crop pests suffer from a lack of efficacy as such programmes most often do not consider the behaviour of predators, which often leads to the uninformed uptake of strategies with unsatisfactory outcomes [20]. For example, Luzon wart frogs (*Fejervarya vittigera*, family Dicroglossidae), native to the Philippines, consume significantly more crop pests (54.1% of diet), while cane toad (*Rhinella marina*, family Bufonidae), introduced as potential bioregulators (89.4% of diet), majorly feed on beneficial arthropod predators [13]. Similarly, rice field frogs (*Minervarya* sp., family Dicroglossidae), a common frog in Southeast Asian agricultural landscapes, feed on beneficial arthropods rather than crop pests, especially at low density [9]. Thus, predators’ response towards prey, diet breadth, prey specificity, prey preference and suitability, searching behaviour, prey recognition (learning), movement, physiology, cognition and home range needs to be considered for developing informed strategies for sustainable pest management. Interestingly, these traits are influenced by foraging modes, i.e. ambush and active foraging [21]. Therefore, we suggest harnessing the diversity in foraging modes of potential pest predators to make pest management both flexible and targeted at the same time. We use amphibians and reptiles as model pest regulators in this study due to the benefits of diverse foraging modes as well as their generalist feeding behaviour that includes a wide range of arthropods many of which are crop pests [22]. Additionally, being generalists, amphibians and reptiles can utilize various resources and tolerate disturbed habitats such as agricultural land uses [23].

Reptiles belonging to the Agamidae, Iguanidea and Cordylidae families are ambush foragers (also known as sit-and-wait foragers), whereas lizards belonging to the superfamily Varanoidea are active foragers (also known as wide foragers) [24]. In amphibians, *Fejervarya* sp. has an ambush foraging mode [25], while Microhylidae generally actively search for prey [26]. We highlight foraging-related traits that are significantly different in the two types of foragers such as movement, learning, memory, sensory faculties and response to prey salience such as size, mobility and occurrence to understand their differential impact on pest regulatory efficiency. Additionally, we also suggest intensification in agriculture as a factor affecting the ecosystem service provisioning by farmland herpetofauna by causing behavioural shifts that can result in either selecting one foraging mode over another or favouring species with mixed foraging strategies.

## 3. Predator movement and its impact on pest regulation

One primary feature that sets apart the two foraging modes is movement. Active forager, such as the green-and-black poison dart frog (*Dendrobates auratus*, family Dendrobatidae), has a foraging velocity of 20 body lengths min^−1^ with a capture rate of 2 prey min^−1^ [27]. In contrast, an ambush forager, the coqui frog (*Eleutherodactylus coqui*, family Eleutherodactylidae), has a maximum foraging velocity of 0.5 body lengths min^−1^ with an attempt rate of only 0.025 prey min^−1^ [28]. Over a 12h activity period, movement of green-and-black poison dart frogs of 2.8 cm snout–vent length is 6000 body lengths, whereas for coqui frogs of similar size, the movement is restricted to 80 body length [28]. Understandably, the estimated daily energy expenditure and the metabolism required by these two foragers are expected to be different. Active foraging Chihuahuan spotted whiptail lizard (*Cnemidophorus exsanguis*, family Teiidae) spends 1.5 times more energy while foraging than the Yarrow’s spiny lizard (*Scleroporus jarrovi,* family Phrynosomatidae), an ambush forager [29]. This is reflected in their prey intake. Food intake by the active forager western whiptail lizard (*Cnemidophorus tigris*, family Teiidae; *ca* 16 g) and the ambush forager zebra-tailed lizard (*Callisaurus draconoides*, family Phrynosomatidae; *ca* 9 g) is different by 374 g (with 426 and 52 mg of the dry mass of arthropod intake, respectively), resulting in a gain of 7.2 kJ of energy for active foragers compared with only 900 J for ambush foragers. Therefore, there is almost a fivefold difference in energy requirement between an active and ambush predator of the same weight. The same variation has been documented in another active forager, the bushveld lizard (*Heliobolus lugubris*, family Lacertidae), in comparison with its syntopic ambush species, the common sand lizard (*Pedioplanis lineoocellata,* family Lacertidae) [30]. Thus, with energy requirements being significantly higher in active foragers, they have the potential to regulate pests up to fivefold more than ambush foragers.

**Figure 1.**
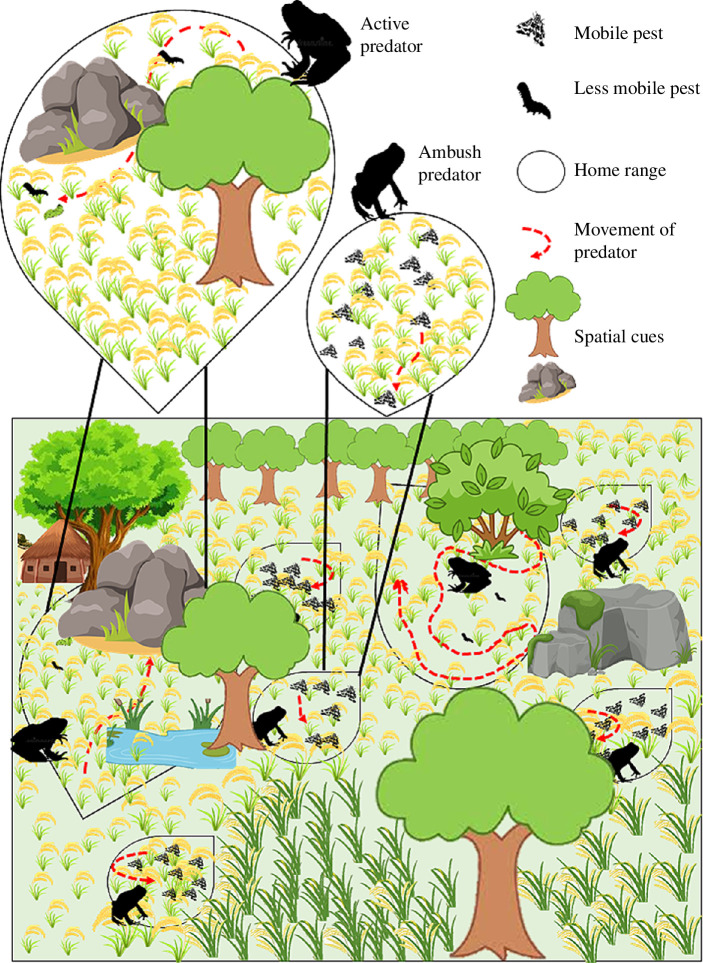
Overview of the concept showing the difference in foraging mode between active and ambush predators impacting biological pest regulation (with amphibians as the model bioregulator). Home range of active predators is larger allowing more movement, greater reliance on landscape cues and active hunting of mobile prey. Ambush predators have a small home range as they move less, need fewer landscape cues and target more mobile prey. Fecundity of active predators is less compared with the ambush predators indicated by the number of predators in the crop field. Movement is indicated by red arrows. Image components are not to scale.

The movement of predators influences their home ranges also potentially influencing their feeding behaviour. Home range sizes are determined by mating strategy and are smaller in ambush predators, while home range sizes are determined by food requirements [31] and are larger in active foragers [32]. Thus, active foragers cover a wider area due to the greater mean speed and a larger home range [33]. Due to their high mobility and the cost of carrying eggs, active foragers tend to carry small egg clutches [34]. Thus, the population size of active predators is generally lower than that of ambush predators and bioregulatory strategies should integrate such information to optimize the benefits [35] (figure 1). For a pest regulator to be efficient, it has to be present in large enough numbers; however, population size needs to be maintained at a threshold, especially for generalist species beyond which the effects will disrupt the balance between ecosystem service and disservice. For generalist predators, the antagonistic effect on biological control is well known, especially because of diffused predation pressure [36,37]. Considering lower dispersal in ambush predators, a highly concentrated population can lead to competition in resource use [38]. Additionally, ambush predators use visual cues originating from prey movement for foraging and predatory arthropods are more agile than herbivore pests [39]. Whereas, in active foragers, individuals being more mobile, the chances of individuals dispersing from concentrated population are higher than ambush predators. However, both active and ambush predators will eventually disperse based on prey abundance, ease of capturing prey and success rate [40]. Further, population size below a threshold in an ambush predator has been shown to shift predation pressure from the crop pests to the predatory arthropods as they are comparatively easier to forage on [9]. Additionally, predation pressure can also impact population size. Higher movement in the active predators attracts predatory risks, while ambush predators sit and wait with short bursts of movement to avoid predatory risks [41]. Such a predatory impact on the overall population of the foragers can significantly influence pest regulation. Therefore, maintaining a threshold population size of pest predators is crucial to sustaining the balance in the ecosystem. This requires the implementation of conservation strategies at the local farmland level as well as the landscape scale to maintain predator population at a certain threshold that benefits bioregulation. Additionally, a regular population size estimate of predators (both vertebrates and arthropods) and crop pests is required. Further, more in-depth research into the various aspects of intraguild predation and the impact of varying predator density on the balance between ecosystem service and disservice can benefit the decision-making regarding potential pest predator. In the case of a highly concentrated pest predator population, pest management programmes can develop strategies that aid the dispersal of the foragers by either actively shifting a part of the population to adjacent non-crop vegetation or by maintaining hedgerows and non-target crop patches. Such vegetation provides diverse prey choice, allowing movement between patches, providing microhabitats, thus attracting and sustaining the predator population while also enabling their movement to crop patches during pest infestation [33,34].

## 4. Predator cognition and its influence on pest regulation

Amphibians and reptiles feed on arthropods belonging to the orders Coleoptera, Diptera, Hemiptera, Hymenoptera, Lepidoptera, Neuroptera, Psocoptera, Isoptera, Thysanoptera, Araneomorphae, Annelida, Blattodea, Decapoda, Dermaptera, Hygrophila, Spirobolida, Trichoptera, Araneae and Orthoptera [6]. Considering the high diversity in arthropod pests, there is significant variation in traits such as size (beetles versus grasshoppers), colour (pentatomid bugs versus grasshoppers), occurrence (aggregated termites versus solitary crickets), movement (moths versus caterpillar), sound (moths versus crickets), taste, odour, as well as infestation type in potential pest prey. These variations necessitate the use of diverse cognitive faculties by the predators.

### 4.1. Response to prey salience and its influence on pest regulation

Foraging mode influences prey choice in predators [27]. Therefore, harnessing the cognitive traits underlying the quality and quantity discrimination abilities of active and ambush predators based on the mobility, size and distribution of prey can help to improve the efficiency of pest regulation. The foraging theory predicts that active foragers prey on sedentary food, whereas ambush foragers capture active and mobile prey [27]. For instance, active foragers such as Bufonid toads and Dendrobatid frogs feed on almost any arthropods, while ambush foragers such as Eleutherodactylidae mostly feed on insects of the orders Diptera, Orthoptera and Coleoptera [27]. In reptiles as well, ambush snakes specialize in active foraging lizards, and active foraging snakes predominantly prey on lizards that rely on ambush foraging [27]. Therefore, sit-and-wait or ambush foraging will be favoured by selection when high-value preys are mobile, and active foraging will be favoured when they are not. Additionally, foraging mode also determines prey size [42], and there is an existing ‘large meal versus small meal’ feeding hypothesis between ambush and active foragers [43]. Gape-limited ambush foraging snakes [44] and amphibians [45] feed on large-bodied prey, while active foragers feed on small-bodied prey. For instance, actively foraging amphibians such as Megophyrids [46] and Bufonids [47] feed on small ants and termites. Active foragers have slender bodies for speed that allow them to capture prey quickly and digest smaller meals easily. In contrast, ambush predators rely on stealth, with squat bodies and broad heads to ingest larger meals. However, this hypothesis may not always be true, and further research is required for a better understanding of how predation mode influences prey selectivity in foragers. Ambush predators encounter prey less frequently; therefore, they cannot be as selective as active foragers and thus should have a broader diet with varying meal sizes [43]. In addition to that, based on prey distribution, the contrary idea is that ambush predators will prefer small-bodied prey when they occur in clumped distributions, while active foragers will prefer large prey with less concentrated distributions [48]. Therefore, prey mobility is an important cue, specific for the foraging modes, while prey size, i.e. small size for active and large size for ambush foragers, shows flexibility. Understanding these dynamics is crucial for effective pest management. In small-scale farming, with high pest diversity and mobility and with either low or high local pest density, a higher proportion of ambush predators may be advantageous due to their opportunistic nature [41]. Conversely, in large-scale farming, more active foragers may ensure better spatial pest regulation and balanced predation pressure between pests based on occurrence patterns. This strategy leverages the strengths of each predator type for targeted, context-dependent pest control, enhancing overall effectiveness. However, further research is required to understand the fundamentals of predator’s response to diverse prey salience to exploit the foraging efficiency of pest predators maximally.

### 4.2. Diverse sensory faculties in predators and influence on pest regulation

Two of the primary faculties that reptiles and amphibians rely on to locate and identify food are visual and olfactory cues [49]. However, the sensory faculty predominantly put into action depends on the foraging mode. Actively foraging lizards (Scleroglossa, Gekkota, Lacertoidea, Scincoidea and Anguimorpha) and amphibians (Microhylid) are highly chemically oriented, while visual acuity in ambush predators (Iguanidae) is a superior faculty to olfaction [25,50,51]. For instance, the oriental garden lizard (*Calotes versicolor,* family Agamidae) is an ambush predator that does not respond to immobile prey or prey chemicals, but only to movement in prey [52]. The kind of sensory faculty that predators rely on, therefore, has a significant influence on their pest regulatory role [6]. Crop pests are cryptic in nature [53], exhibit behavioural predatory avoidance and use behavioural defences like crypsis, refuge-seeking [54], etc. Therefore, if visual cues are primarily used to locate prey, there is a high probability that cryptic pests will evade biocontrol strategies. However, active foragers can be utilized to suppress cryptic/immobile pests because they actively hunt through olfaction, while ambush predators can be utilized to target the mobile adult life-history stages of the pests. Therefore, active and ambush foragers can be strategically used to target specific life-history stages of crop pests thus optimizing the bioregulation by these natural predators.

Alongside the fact that different foraging modes allow predators to respond to specific prey salience such as movement, size and prey occurrence, there are additional factors that lead to successful foraging decision-making such as prey abundance, competition in a crop patch, habitat quality and predatory risks. Although a lot is known about optimal foraging strategies in animals, integrating this concept into biological pest regulation is still in its infancy [55–57]. To ensure optimal efficiency of pest regulation by active and ambush predators, more fundamental research is required, but there is immense potential in the concept, and without expanding our knowledge on the response of a predator to its prey (i.e. what cues in the prey a predator uses to forage), results from biological pest regulation may remain inconclusive [58].

## 5. Spatial cognition in predators and its influence on pest regulation

Reptiles and amphibians learn about fitness-enhancing features within their home range [59] and site-specific cues help them to create a cognitive map, i.e. a spatial representation of environmental cues [60]. Spatial learning is important for foraging [61] and attaching memory to fitness-enhancing features in a cognitive map is beneficial when food is patchily distributed in space as predators can navigate based on previous experience avoiding spending energy prospecting sites in every visit [62]. According to the spatial adaptation model, which suggests that correlations between spatial ability and spatially demanding niches are a consequence of selection for navigational demands [63], active foragers are more proficient than ambush predators and have better spatial cognition [64]. This is understandable because of the larger home range and greater mobility in the active foragers that required better cognitive skills. Additionally, active foragers spend time and energy searching for prey and are quick to decide on leaving a non-rewarding site to forage where food is more abundant [65]. Therefore, active foragers are more efficient in searching for rewarding foraging patches, a behaviour enabled by spatial cues [66]. In contrast, ambush predators invest less time and energy in searching for prey [67], because they sit and wait for prey, although they will eventually switch to sites where prey is more abundant. Being less mobile, the reliance on spatial cues is less in ambush foragers.

The alternative argument to the spatial adaptation model is the pliancy model. This model states that the ecological demand selects for behavioural flexibility and may not be predisposed [63]. For instance, active foraging may select for behavioural flexibility and require the memory of complex associations [68] to navigate in a habitat. For instance, Bosc’s fringe-toed lizard (*Acanthodactylus boskianus*, family Lacertidae), an active forager, and the Nidua fringe-fingered lizard (*Acanthodactylus scutellatus,* family Lacertidae), a congeneric ambush forager [63], show no significant difference in performance in a spatial task. This finding supports the pliancy model that ecological demands dictate behavioural flexibility rather than selection. Thus, more research is required to disentangle the two theoretical hypotheses to test their potential and implications in pest management.

Agricultural lands are dynamic landscapes that experience rapid change across seasons, crop types and agricultural practices, consequently altering local cues such as distinct landscape features, colour, position of landscape components, etc. Amphibians and reptiles can use learned spatial cues based on experience [69,70]. The use of beacon-guidance strategy (guidance learning using proximate cues), egocentric cues (local views or snapshot mechanisms) and even allocentric cues (learning the location of a goal by encoding its spatial relationships with a number of distant landmarks to form a map-like representation or place learning) is well understood in amphibians and reptiles [60,71]. Pest management can make use of the spatial learning flexibility in both the foragers by adding components in the farmland that enable and reinforce their learning about rewarding crop patches. Crop fields with a higher proportion of ambush foragers can adopt a beacon-guidance strategy or use more local cues within patches such that individuals can recognize and remember their ambush sites as ambush predators are known to perform better when they are positioned within foraging patches [41]. The presence of guidance such as a distinct geometric structure or even a natural rock of the same colour, size and texture can be used as an indicator of the rewarding crop patch [40]. Being more efficient in learning cues, active predators can be better at prospecting the surroundings and rapidly forming memories to remember the location of a pest-infested crop patch [63]. Therefore, for active foragers, using spatial cues (landscape cues) such as trees or perennial natural habitats near pest-infested crop patches might prove to be beneficial, as based on their spatial needs, active foragers can be expected to be efficient in using such allocentric cues. Additionally, maintaining a stable habitat such as a similar position of crop patches can help to keep the landmarks, distal cues and geometric cues more stable, allowing both active and ambush foragers to develop a stable map for a reliable association of the features in a crop patch [72,73]. The idea of harnessing spatial cognition in active and ambush foragers requires more research for effectively adopting the strategy to improve their pest regulation.

## 6. Agricultural intensification and its effect on predators’ bioregulatory efficiency

Considering the growing body of evidence on the negative effects of chemical control of crop pests on ecosystem and biodiversity, the need for biological control has never been so critical [9,74]. Herpetofauna has been tested in classical [14], augmentative [15] and conservation biocontrol [9]. Classical biological control in which exotic species are released in new environments to regulate pest population has shown a negative influence on the ecosystem and its associated diversity (cane toads in Australia released as a potential pest regulator are now a national threat [75]). Augmentative biocontrol has the potential to make pest management more targeted by selecting and releasing active and/or ambush predators based on the spatial scale of the farmland, crop type and the pest infestation level, and the specific prey salience that can bring out the optimum response from the pest predators. However, such a strategy will miss out on utilizing the full potential of the diversity of the foraging modes. Agricultural intensification has presented a dual challenge of mitigating crop pests while threatening the naturally occurring diversity, compromising the services they provide [76]. Conservation biological control, which harnesses strategies to preserve the population of natural predators already in the environment, is the most sought strategy considering the targeted effect on pest regulation and the zero non-target impact on the ecosystem [77]. This strategy necessitates the uptake of information on diverse aspects of the two foraging modes complemented by the redesigning of farmlands to allow for the optimal expression of the foraging traits. Thus, utilizing the diversity in both the foraging modes and the cognitive underpinning of decision-making could be the next step for advancing pest regulation at the same time as protecting their diversity in farmlands.

**Figure 2.**
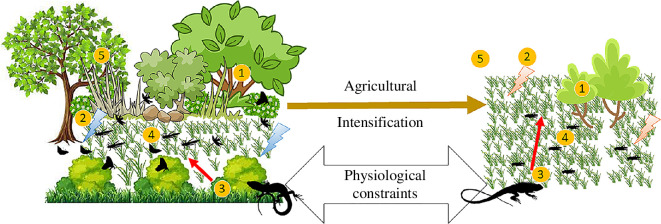
Probable landscape changes due to agricultural intensification that can alter foraging modes (with reptile as the model bioregulator). Changes in the landscape due to the intensification of agriculture (left to right) causing alterations in available retreats (1), cooler versus hotter microhabitat (2), low versus high visual field (3), lower versus higher prey density and prey types (4) and more versus fewer landscape cues (5) that can select for altered foraging modes posing physiological constraints on the foragers.

Additionally, amphibians and reptiles also exhibit flexibility in their foraging modes which can be beneficial to restore the ecosystem services in the dynamic and unstable agricultural landscapes. Agricultural land uses cause alterations in the structure and composition of vegetation and ground cover [78]. Amphibians and reptiles can switch foraging modes within species and between contexts [79], especially in response to microgeographic variability due to habitat alteration [80]. The construction of walls in farmlands caused behavioural shifts in the Aegean wall lizards (*Podarcis erhardii*, family Lacertidae), which is essentially an active predator feeding on sedentary prey. However, they showed a higher propensity to jump to catch active prey in habitats with human-built walls [80], a trait generally displayed in ambush predators. The shift from active to ambush foraging in *P. erhardii* also induced phenotypic changes including the size of hind limbs, forelimbs and head structure. Such phenotypic shifts can also be induced by other triggers. For instance, active foraging Be’er Sheva fringe-fingered lizard (*Acanthodactylus beershebensis,* family Lacertidae) switched to ambush predation as a response to kestrels causing a shift in diet towards smaller and more active insects [81]. The ocellated sand lizard (*Pedioplanis lineoocellata*, family Lacertidae) shifts from ambush to active foraging in response to overgrazing in agricultural lands [82]. This switch in foraging is the result of a lower prey abundance and results in the species spending more time moving to cover larger distances, over unfavourable habitats in degraded landscapes. Modification of landscape structure, habitat, ground cover, predation pressure and prey availability are among the consequences of intensifying agriculture that can drive shifts in the foraging behaviour of the resident herpetofauna although to an unknown extent, with variable consequences (figure 2, table 1).

**Table 1.** Directionality of changes in foraging modes due to an increase in agricultural intensification, with putative rationales and constraints to the shift in foraging modes.

behavioural shift due to agricultural intensification	rationales favouring the shift	major constraints to the shift
active to ambush	lack of retreats will favour individuals sitting and waiting for pests	physiological constraints of the predator
	lower dependence on spatial cues	only one particular pest type will be targeted based on movement (mostly mobile prey)
	increased visual field	
	easy pick-up of prey movement	
	clumped distribution of prey	
ambush to active	lack of suitable ambush sites	landscape resistance
	change in pest density	physiological constraints
	prey occurrence in less concentration	only one particular prey type is targeted (less mobile) based on olfaction
		less spatial cues to navigate
active/ambush to mixed	suitably adapted to change according to habitat structure	no major physiological constraint therefore, show flexibility in foraging
	target different crop types based on availability	

Behavioural flexibility is a buffer mechanism enabling species to survive and rapidly adapt to degraded and unfavourable habitats [82]. However, there are physical and physiological constraints to switching foraging mode [83]. ‘Landscape resistance’ or ‘landscape impedance’ (a measurement of how landscape structure influences movement patterns in animals) resulting from habitat modification can alter the thermal regime (thermal properties of microhabitat materials that enable proper exchanges of heat and moisture between the animal and the ground surface) of individuals as intensive agriculture is more homogeneous in structural components, with lesser natural vegetation and more open space [84]. Degraded habitat can deter the movement of amphibians and reptiles due to a higher chance of desiccation and select for predators that rely on visual cues like ambush predators rather than active foragers [85]. However, physiological constraints may prevent species from shifting foraging mode as seen in the ambush predator *E. coqui*, with its low aerobic capacity, being physiologically incapable of assuming the same active foraging mode as that of *D. auratus*. Likewise, a low capacity for anaerobic glycolysis in Dendrobatids would make them slow and inefficient as ambush predators [28]. In contrast, some lizards and amphibians exhibit both active and ambush foraging [86]. The two-coloured oval frogs (*Elachistocleis bicolor*, family Microhylidae) actively search for ant trails, and upon encountering them individuals sit and wait for ants to pass and forage [24]. Among lizard families, some Lacertidae [87], Gekkonidae [88], Pygopodidae [89] and Scincidae [90] exhibit substantial variation in foraging modes, including species that exhibit intermediate, or mixed foraging modes [33]. Thus, it is possible for different degrees of intensive agriculture to support species that exhibit flexibility in the foraging modes [83] (figure 2). Therefore, pest management practitioners need to redesign farmlands such that both the foraging traits are conserved in a landscape rather than selecting one over the other. Developing hedgerow landscapes, a form of ecological intensification that harnesses ecosystem services to sustain agricultural production while minimizing adverse effects of agriculture on the ecosystem*,* is an effective strategy. These landscapes combine natural and anthropized habitats, enhancing biodiversity and ecosystem services such as pest control [91]. They also offer thermal buffering against climate change [92]. Hedgerows provide essential microhabitats for amphibians and reptiles, aiding in thermal and hydro-regulation, food supply and refuge [93,94]. This redesign could significantly improve the conservation of the ecosystem services rendered by the active and ambush foragers while also conserving the species in the farmlands. Additionally, agriculture has been predicted to be the most threatened land use category due to climate warming [95], and amphibians and reptiles are among the most vulnerable group of species. However, reptiles experiencing early life increase in thermal environment develop better foraging skills [96]. Therefore, it is worth exploring the impact of climate warming on foraging behaviour in amphibians and reptiles to understand their ability to cope with the changing thermal regime and how agriculture can benefit from it.

## 7. Conclusion

The overall aim of this work is to urge the uptake of animal behaviour and cognition in pest management science (figure 1). From both ecological and behavioural aspects, active foragers are more efficient in pest regulation due to higher energetic needs requiring them to actively scan the environment, resulting in a prey capture rate higher than that of an ambush forager with similar body size. The two foraging modes generally rely on different sensory faculties that can be harnessed to target different life-history stages of crop pests. However, population size of the pest predators is a crucial consideration that can impact the overall success of a pest management programme. Further, transformation of agricultural lands can threaten the ecosystem services of the resident natural pest predators by causing behavioural shifts through changes in prey base, thermal regime, microhabitat structure and refuge availability, resulting in the selection of a specific foraging mode while eliminating the other. Therefore, we emphasize the need for adaptive management practices in response to environmental changes, focusing on the physiological and behavioural responses of species to restore ecological services effectively as well as maintain the threshold population of natural resources [97,98].

Our work should spark a re-evaluation of the current approach to biological pest regulation. The benefits of using vertebrates in pest regulation, especially amphibians and reptiles, lie in their flexibility, longevity and specificity in target pest type and spatial extent of pest regulation based on foraging modes. Looking forward, we believe pest management science should move towards utilizing our understanding of the cognitive traits and mechanisms that underlie the foraging decision-making in pest predators. Our perspective on using foraging modes to guide bioregulation strategies should serve as a preface for designing experimental studies that can contribute to keep pest management evolving.

## Data Availability

This article has no additional data.

## References

[1] Isman MB . 2019 Challenges of pest management in the twenty first century: new tools and strategies to combat old and new foes alike. Front. Agron. **1** , 00002. (10.3389/fagro.2019.00002)

[2] Dudley N , Alexander S . 2017 Agriculture and biodiversity: a review. Biodiversity **18** , 45–49. (10.1080/14888386.2017.1351892)

[3] Van Leeuwen T , Van Pottelberge S , Nauen R , Tirry L . 2007 Organophosphate insecticides and acaricides antagonise bifenazate toxicity through esterase inhibition in Tetranychus urticae. Pest Manag. Sci. **63** , 1172–1177. (10.1002/ps.1453) 17880043

[4] Shikano I , Isman MB . 2009 A sensitive period for larval gustatory learning influences subsequent oviposition choice by the cabbage looper moth. Anim. Behav. **77** , 247–251. (10.1016/j.anbehav.2008.08.033)

[5] Korányi D , Egerer M , Rusch A , Szabó B , Batáry P . 2022 Urbanization hampers biological control of insect pests: a global meta-analysis. Sci. Total Environ. **834** , 155396. (10.1016/j.scitotenv.2022.155396) 35460770

[6] Ghosh D , John EA , Wilkinson A . 2023 Clever pest control? The role of cognition in biological pest regulation. Anim. Cogn. **26** , 189–197. (10.1007/s10071-022-01731-4) 36526865 PMC9877098

[7] Ives AR , Cardinale BJ , Snyder WE . 2005 A synthesis of subdisciplines: predator–prey interactions, and biodiversity and ecosystem functioning. Ecol. Lett. **8** , 102–116. (10.1111/j.1461-0248.2004.00698.x)

[8] Lindell C , Eaton RA , Howard PH , Roels SM , Shave ME . 2018 Enhancing agricultural landscapes to increase crop pest reduction by vertebrates. Agric. Ecosyst. Environ. **257** , 1–11. (10.1016/j.agee.2018.01.028)

[9] Ghosh D , Basu P . 2023 Does agricultural intensification impact pest regulation service by frogs in a natural multi-trophic system? Perspect. Ecol. Conserv. **21** , 216–223. (10.1016/j.pecon.2023.06.002)

[10] Khatiwada JR , Ghimire S , Paudel Khatiwada S , Paudel B , Bischof R , Jiang J , Haugaasen T . 2016 Frogs as potential biological control agents in the rice fields of Chitwan, Nepal. Agric. Ecosyst. Environ. **230** , 307–314. (10.1016/j.agee.2016.06.025)

[11] Monagan IV , Morris JR , Davis Rabosky AR , Perfecto I , Vandermeer J . 2017 Anolis lizards as biocontrol agents in mainland and Island agroecosystems. Ecol. Evol. **7** , 2193–2203. (10.1002/ece3.2806) 28405283 PMC5383488

[12] Hirai T . 2007 Diet composition of Hyla japonica in soybean fields: is a euryphagous predator useful for pest management? Jpn. J. Appl. Entomol. Zool. **51** , 103–106. (10.1303/jjaez.2007.103)

[13] Shuman-Goodier ME , Diaz MI , Almazan ML , Singleton GR , Hadi BAR , Propper CR . 2019 Ecosystem hero and villain: native frog consumes rice pests, while the invasive cane toad feasts on beneficial arthropods. Agric. Ecosyst. Environ. **279** , 100–108. (10.1016/j.agee.2019.04.008)

[14] Teng Q , Hu XF , Luo F , Cheng C , Ge X , Yang M , Liu L . 2016 Influences of introducing frogs in the paddy fields on soil properties and rice growth. J. Soil. Sediment. **16** , 51–61. (10.1007/s11368-015-1183-6)

[15] Fang K , Yi X , Dai W , Gao H , Cao L . 2019 Effects of integrated rice-frog farming on paddy field greenhouse gas emissions. Int. J. Environ. Res. Public Health **16** , 1930. (10.3390/ijerph16111930) 31159212 PMC6603866

[16] Losey JE , Vaughan M . 2006 The economic value of ecological services provided by insects. Bioscience **56** , 311. (10.1641/0006-3568(2006)56[311:TEVOES]2.0.CO;2)

[17] Ceron K , Santana DJ , Pires MM . 2024 The economic risk of the losses in pest control as frogs decline. EcoEvoRxiv. (10.32942/X2SP79)

[18] Wasko DK , Sasa M . 2012 Food resources influence spatial ecology, habitat selection, and foraging behavior in an ambush-hunting snake (Viperidae: Bothrops asper): an experimental study. Zool. Jena. **115** , 179–187. (10.1016/j.zool.2011.10.001) 22440190

[19] Jeffers P . 2021 Biological Control Strategies in Integrated Pest Management (IPM) Programs. Land-Grant Press, Clemson University, South Carolina. See https://lgpress.clemson.edu/publication/biological-control-strategies-in-integrated-pest-management-ipm-programs/.

[20] Howarth FG . 1991 Environmental impacts of classical biological control. Annu. Rev. Entomol. **36** , 485–509. (10.1146/annurev.en.36.010191.002413)

[21] Lisiecki C . 2019 Efficacy of the Aegean wall lizard (Podarcis erhardii) as a potential biological control agent in Mediterranean agroecosystems. See http://deepblue.lib.umich.edu/handle/2027.42/148803.

[22] Valencia-Aguilar A , Cortés-Gómez AM , Ruiz-Agudelo CA . 2013 Ecosystem services provided by amphibians and reptiles in neotropical ecosystems. Int. J. Biodivers. Sci. Ecosyst. Serv. Manage. **9** , 257–272. (10.1080/21513732.2013.821168)

[23] Nordberg EJ , Schwarzkopf L . 2019 Reduced competition may allow generalist species to benefit from habitat homogenization. J. Appl. Ecol. **56** , 305–318. (10.1111/1365-2664.13299)

[24] Perry G . 2007 Movement patterns in lizards: measurement, modality, and behavioral correlates. Lizard Ecol. Evol. Conseq. Forag. Mode. 13–48. (10.1017/CBO9780511752438.003)

[25] Inger RF , Szarski H , Kollros JJ , Duellman WE , Trueb L . 1986 Biology of amphibians. Copeia **1986** , 549. (10.2307/1445022)

[26] López JA , Antoniazzi CE , Lorenzón R , Ghirardi R . 2017 Spatio-temporal patterns of foraging and feeding behavior of Elachistocleis bicolor (Anura: Microhylidae). Cal. **39** , 345–353. (10.15446/caldasia.v39n2.63397)

[27] Huey RB , Pianka ER . 1981 Ecological consequences of foraging mode. Ecology **62** , 991–999. (10.2307/1936998)

[28] Taigen TL , Pough FH . 1985 Metabolic correlates of Anuran behavior. Am. Zool. **25** , 987–997. (10.1093/icb/25.4.987)

[29] Brown TK , Nagy KA . 2007 Lizard energetics and the sit-and-wait vs. wide-foraging paradigm. Lizard Ecol. 120–140. (10.1017/CBO9780511752438.006)

[30] Nagy KA , Huey RB , Bennett AF . 1984 Field energetics and foraging mode of Kalahari lacertid lizards. Ecology **65** , 588–596. (10.2307/1941421)

[31] Verwaijen D , Damme RV . 2008 Wide home ranges for widely foraging lizards. Zoology (Jena). **111** , 37–47. (10.1016/j.zool.2007.04.001) 17997294

[32] Cooper WE , Vitt LJ , Caldwell JP , Fox SF . 2001 Foraging modes of some american lizards: relationships among measurement variables and discreteness of modes. Herpetologica **57** , 65–76.

[33] Cooper Jr WE , Whiting MJ . 2000 Ambush and active foraging modes both occur in the scincid genus mabuya. Copeia **2000** , 112–118. (10.1643/0045-8511(2000)2000[0112:AAAFMB]2.0.CO;2)

[34] Vitt LJ , Price HJ . 1982 Ecological and evolutionary determinants of relative clutch mass in lizards. Herpetologica **38** , 237–255.

[35] Suárez-Varón G , Suárez-Rodríguez O , Granados-González G , Villagrán-Santa Cruz M , Gribbins KM , Cortez-Quezada D , Hernández-Gallegos O . 2019 Relative clutch mass of Basiliscus vittatus Wiegmann, 1828 (Squamata, Corytophanidae): female morphological constraints. Herpetozoa **32** , 211–219. (10.3897/herpetozoa.32.e35910)

[36] Perez-Alvarez R , Nault BA , Poveda K . 2019 Effectiveness of augmentative biological control depends on landscape context. Sci. Rep. **9** , 8664. (10.1038/s41598-019-45041-1) 31209256 PMC6572857

[37] Wells K . 2007 The ecology & behavior of amphibians. Chicago, IL, USA: Bibliovault OAI Repository, The University of Chicago Press. (10.7208/chicago/9780226893334.001.0001)

[38] Stephens DW . 2008 Decision ecology: foraging and the ecology of animal decision making. Cogn. Affect. Behav. Neurosci. **8** , 475–484. (10.3758/CABN.8.4.475) 19033242

[39] Shahzad Ahmed K , Majeed M , Haidary A , Haider N . 2016 Integrated pest management tactics and predatory coccinellids: a review. J. Entomol. Zool. Stud. **4** , 591–600.

[40] González-Bernal E , Brown GP , Cabrera-Guzmán E , Shine R . 2011 Foraging tactics of an ambush predator: the effects of substrate attributes on prey availability and predator feeding success. Behav. Ecol. Sociobiol. **65** , 1367–1375. (10.1007/s00265-011-1147-9)

[41] Scharf I . 2024 Search patterns, resource regeneration, and ambush locations impact the competition between active and ambush predators. Ann. N. Y. Acad. Sci. **1536** , 122–134. (10.1111/nyas.15169) 38861340

[42] Pough FH , Groves JD . 1983 Specializations of the body form and food habits of snakes. Am. Zool. **23** , 443–454. (10.1093/icb/23.2.443)

[43] Glaudas X , Glennon KL , Martins M , Luiselli L , Fearn S , Trembath DF , Jelić D , Alexander GJ . 2019 Foraging mode, relative prey size and diet breadth: a phylogenetically explicit analysis of snake feeding ecology. J. Anim. Ecol. **88** , 757–767. (10.1111/1365-2656.12972) 30828806

[44] Secor SM , Diamond JM . 2000 Evolution of regulatory responses to feeding in snakes. Physiol. Biochem. Zool. **73** , 123–141. (10.1086/316734) 10801391

[45] Toft CA . 1980 Feeding ecology of thirteen syntopic species of anurans in a seasonal tropical environment. Oecologia **45** , 131–141. (10.1007/BF00346717) 28310947

[46] Toft CA . 1981 Feeding ecology of Panamanian litter anurans: patterns in diet and foraging mode. J. Herpetol. **15** , 139. (10.2307/1563372)

[47] Cundall D , Greene H . 2000 Feeding in snakes. In Feeding: form, function, and evolution in tetrapod vertebrates (ed. K Schwenk ), pp. 293–333. San Diego, CA: Academic Press. (10.1016/B978-012632590-4/50010-1)

[48] Emerson SB . 1976 Burrowing in frogs. J. Morphol. **149** , 437–458. (10.1002/jmor.1051490402) 30257534

[49] Stanger-Hall KF , Zelmer DA , Bergren C , Burns SA . 2001 Taste discrimination in a lizard (Anolis carolinensis, Polychrotidae). Copeia **2001** , 490–498. (10.1643/0045-8511(2001)001[0490:TDIALA]2.0.CO;2)

[50] Vidal N , Hedges SB . 2009 The molecular evolutionary tree of lizards, snakes, and amphisbaenians. C. R. Biol. **332** , 129–139. (10.1016/j.crvi.2008.07.010) 19281946

[51] Hoare JM , Pledger S , Nelson NJ . 2007 Chemical discrimination of food, conspecifics and predators by apparently visually-oriented diurnal geckos, Naultinus manukanus. Herpetologica **63** , 184–192. (10.1655/0018-0831(2007)63[184:CDOFCA]2.0.CO;2)

[52] Ammanna VHF , Saidapur SK , Shanbhag BA . 2014 Prey detection in juveniles of an agamid lizard, Calotes versicolor (Daudin, 1802) (Reptilia: Squamata). Ital. J. Zool. **81** , 155–159. (10.1080/11250003.2013.875600)

[53] Chakraborty S , Tiwari PK , Sasmal SK , Biswas S , Bhattacharya S , Chattopadhyay J . 2017 Interactive effects of prey refuge and additional food for predator in a diffusive predator-prey system. Appl. Math. Model. **47** , 128–140. (10.1016/j.apm.2017.03.028)

[54] Sih A , Moore RD . 1990 Interacting effects of predator and prey behavior in determining diets. In Behavioural mechanisms of food selection (ed. RN Hughes ), pp. 771–796. Berlin, Germany: Springer. (10.1007/978-3-642-75118-9_37)

[55] Davis GH , Crofoot MC , Farine DR . 2022 Using optimal foraging theory to infer how groups make collective decisions. Trends Ecol. Evol. **37** , 942–952. (10.1016/j.tree.2022.06.010) 35842325

[56] Kie JG . 1999 Optimal foraging and risk of predation: effects on behavior and social structure in ungulates. J. Mammal. **80** , 1114–1129. (10.2307/1383163)

[57] Sinervo B . 2007 Optimal foraging theory: constraints and cognitive. See https://www.yumpu.com/en/document/view/18608470/chapter-6-optimal-foraging-theory-constraints-and-cognitive.

[58] Approaches to the Biological Control of Insect Pests . CT.gov - Connecticut’s Official State Website. See https://portal.ct.gov/CAES/Fact-Sheets/Entomology/Approaches-to-the-Biological-Control-of-Insect-Pests.

[59] Olton DS , Weiskrantz L . 1997 The temporal context of spatial memory. Phil. Trans. R. Soc. Lond. B **308** , 79–86. (10.1098/rstb.1985.0011)

[60] Liu Y , Day LB , Summers K , Burmeister SS . 2019 A cognitive map in a poison frog. J. Exp. Biol. **222** , jeb197467. (10.1242/jeb.197467) 31182504

[61] Carazo P , Noble DWA , Chandrasoma D , Whiting MJ . 2014 Sex and boldness explain individual differences in spatial learning in a lizard. Proc. R. Soc. B **281** , 20133275. (10.1098/rspb.2013.3275) PMC3973267 24619443

[62] Bracis C , Gurarie E , Van Moorter B , Goodwin RA . 2015 Memory effects on movement behavior in animal foraging. PLoS One **10** , e0136057. (10.1371/journal.pone.0136057) 26288228 PMC4542208

[63] Day LB , Crews D , Wilczynski W . 1999 Spatial and reversal learning in congeneric lizards with different foraging strategies. Anim. Behav. **57** , 393–407. (10.1006/anbe.1998.1007) 10049480

[64] Beachly WM , Stephens DW , Toyer KB . 1995 On the economics of sit-and-wait foraging: site selection and assessment. Behav. Ecol. **6** , 258–268. (10.1093/beheco/6.3.258)

[65] Brown JS . 1988 Patch use as an indicator of habitat preference, predation risk, and competition. Behav. Ecol. Sociobiol. **22** , 37–47. (10.1007/BF00395696)

[66] Carle T , Horiwaki R , Hurlbert A , Yamawaki Y . 2018 Aversive learning in the praying mantis (Tenodera aridifolia), a sit and wait predator. J. Insect Behav. **31** , 158–175. (10.1007/s10905-018-9665-1) 29628622 PMC5882761

[67] Anderson RA , Karasov WH . 1981 Contrasts in energy intake and expenditure in sit-and-wait and widely foraging lizards. Oecologia **49** , 67–72. (10.1007/BF00376899) 28309450

[68] Whiting MJ , Noble DWA . 2018 Lizards – measuring cognition: practical challenges and the influence of ecology and social behaviour. In Field and laboratory methods in animal cognition (eds N Bueno-Guerra , F Amici ), pp. 266–285. Cambridge, UK: Cambridge University Press. (10.1017/9781108333191.014)

[69] Pašukonis A , Trenkwalder K , Ringler M , Ringler E , Mangione R , Steininger J , Warrington I , Hödl W . 2016 The significance of spatial memory for water finding in a tadpole-transporting frog. Anim. Behav. **116** , 89–98. (10.1016/j.anbehav.2016.02.023) 28239185 PMC5321284

[70] LaDage LD , Cobb Irvin TE , Gould VA . 2017 Assessing spatial learning and memory in small squamate reptiles. J. Vis. Exp. **119** , e55103. (10.3791/55103) PMC5351821 28117775

[71] López J , Gómez Y , Rodríguez F , Broglio C , Vargas J , Salas C . 2001 Spatial learning in turtles. Anim. Cogn. **4** , 49–59. (10.1007/s100710100091)

[72] Sovrano VA , Baratti G , Lee SA . 2020 The role of learning and environmental geometry in landmark-based spatial reorientation of fish (Xenotoca eiseni). PLoS One **15** , e0229608. (10.1371/journal.pone.0229608) 32126075 PMC7053775

[73] Noble DWA , Carazo P , Whiting MJ . 2012 Learning outdoors: male lizards show flexible spatial learning under semi-natural conditions. Biol. Lett. **8** , 946–948. (10.1098/rsbl.2012.0813) 23075525 PMC3497152

[74] Bommarco R , Kleijn D , Potts SG . 2013 Ecological intensification: harnessing ecosystem services for food security. Trends Ecol. Evol. **28** , 230–238. (10.1016/j.tree.2012.10.012) 23153724

[75] Shanmuganathan T *et al* . 2010 Biological control of the cane toad in Australia: a review. Anim. Conserv. **13** , 16–23. (10.1111/j.1469-1795.2009.00319.x)

[76] Waage J . In press Commission on genetic resources for food and agriculture.

[77] Bale JS , van Lenteren JC , Bigler F . 2008 Biological control and sustainable food production. Phil. Trans. R. Soc. B **363** , 761–776. (10.1098/rstb.2007.2182) 17827110 PMC2610108

[78] Buyantuyev A , Wu J . 2010 Urban heat islands and landscape heterogeneity: linking spatiotemporal variations in surface temperatures to land-cover and socioeconomic patterns. Landsc. Ecol. **25** , 17–33. (10.1007/s10980-009-9402-4)

[79] Donihue CM , Brock KM , Foufopoulos J , Herrel A . 2016 Feed or fight: testing the impact of food availability and intraspecific aggression on the functional ecology of an island lizard. Funct. Ecol. **30** , 566–575. (10.1111/1365-2435.12550)

[80] Wild KH , Gienger CM . 2018 Fire‐disturbed landscapes induce phenotypic plasticity in lizard locomotor performance. J. Zool. **305** , 96–105. (10.1111/jzo.12545)

[81] Hawlena D , Pérez-Mellado V . 2009 Change your diet or die: predator-induced shifts in insectivorous lizard feeding ecology. Oecologia **161** , 411–419. (10.1007/s00442-009-1375-0) 19466458

[82] Wasiolka B , Blaum N , Jeltsch F , Henschel J . 2009 Behavioural responses of the lizard Pedioplanis l. lineoocellata to overgrazing. Acta Oecol. **35** , 157–162. (10.1016/j.actao.2008.09.009)

[83] Huey RB , Bennett AF , John-Alder H , Nagy KA . 1984 Locomotor capacity and foraging behaviour of kalahari lacertid lizards. Anim. Behav. **32** , 41–50. (10.1016/S0003-3472(84)80322-X)

[84] Lebrija-Trejos E , Meave JA , Poorter L , Pérez-García EA , Bongers F . 2010 Pathways, mechanisms and predictability of vegetation change during tropical dry forest succession. Perspect. Plant Ecol. Evol. Syst. **12** , 267–275. (10.1016/j.ppees.2010.09.002)

[85] Sahlean TC , Papeș M , Strugariu A , Gherghel I . 2020 Ecological corridors for the amphibians and reptiles in the Natura 2000 sites of Romania. Sci. Rep. **10** , 19464. (10.1038/s41598-020-76596-z) 33173154 PMC7655805

[86] Maneyro R , Rosa ID . 2004 Temporal and spatial changes in the diet of Hyla pulchella (Anura, Hylidae) in Southern Uruguay. Phyllomedusa **3** , 101. (10.11606/issn.2316-9079.v3i2p101-103)

[87] Perry G , Lampl I , Lerner A , Rothenstein D , Shani E , Sivan N , Werner YL . 1990 Foraging mode in lacertid lizards: variation and correlates. Amphib. reptil. **11** , 373–384. (10.1163/156853890X00069)

[88] Arnold EN . 1990 Why do morphological phylogenies vary in quality? An investigation based on the comparative history of lizard clades. Proc. R. Soc. Lond. B **240** , 135–172. (10.1098/rspb.1990.0031) 1972988

[89] Webb JK , Shine R . 1994 Feeding habits and reproductive biology of Australian pygopodid lizards of the genus Aprasia. Copeia **1994** , 390. (10.2307/1446986)

[90] Cooper WE . 1995 Foraging mode, prey chemical discrimination, and phylogeny in lizards. Anim. Behav. **50** , 973–985. (10.1016/0003-3472(95)80098-0)

[91] Staley JT , Amy SR , Adams NP , Chapman RE , Peyton JM , Pywell RF . 2015 Re-structuring hedges: rejuvenation management can improve the long term quality of hedgerow habitats for wildlife in the UK. Biol. Conserv. **186** , 187–196. (10.1016/j.biocon.2015.03.002)

[92] Vanneste T *et al* . 2020 Contrasting microclimates among hedgerows and woodlands across temperate Europe. Agric. For. Meteorol. **281** , 107818. (10.1016/j.agrformet.2019.107818)

[93] Arntzen JW , Abrahams C , Meilink WRM , Iosif R , Zuiderwijk A . 2017 Amphibian decline, pond loss and reduced population connectivity under agricultural intensification over a 38 year period. Biodivers. Conserv. **26** , 1411–1430. (10.1007/s10531-017-1307-y)

[94] Blouin-Demers G , Weatherhead PJ . 2001 Thermal ecology of black rat snakes (Elaphe obsoleta) in a thermally challenging environment. Ecology **82** , 3025–3043. (10.1890/0012-9658(2001)082[3025:TEOBRS]2.0.CO;2)

[95] Raza A , Razzaq A , Mehmood SS , Zou X , Zhang X , Lv Y , Xu J . 2019 Impact of climate change on crops adaptation and strategies to tackle its outcome: a review. Plants **8** , 34. (10.3390/plants8020034) 30704089 PMC6409995

[96] Siviter H , Deeming DC , Wilkinson A . 2019 Egg incubation temperature influences the growth and foraging behaviour of juvenile lizards. Behav. Processes **165** , 9–13. (10.1016/j.beproc.2019.06.003) 31170461

[97] Östman Ö , Ekbom B , Bengtsson J . 2003 Yield increase attributable to aphid predation by ground-living polyphagous natural enemies in spring barley in Sweden. Ecol. Econ. **45** , 149–158. (10.1016/S0921-8009(03)00007-7)

[98] Gallai N , Salles JM , Settele J , Vaissière BE . 2009 Economic valuation of the vulnerability of world agriculture confronted with pollinator decline. Ecol. Econ. **68** , 810–821. (10.1016/j.ecolecon.2008.06.014)

